# The mechanisms and significance of up‐regulation of RhoB expression by hypoxia and glucocorticoid in rat lung and A549 cells

**DOI:** 10.1111/jcmm.12809

**Published:** 2016-02-24

**Authors:** Gao‐Xiang Huang, Xiao‐Yu Pan, Yi‐Duo Jin, Yan Wang, Xiao‐Lian Song, Chang‐Hui Wang, Yi‐Dong Li, Jian Lu

**Affiliations:** ^1^ Department of PathophysiologyThe Second Military Medical UniversityShanghaiChina; ^2^ Department of Respiratory MedicineShanghai Tenth People's HospitalTongji UniversityShanghaiChina

**Keywords:** cell survival, dexamethasone, hypoxia, RhoB

## Abstract

Small guanosine triphosphate (GTP)‐binding protein RhoB is an important stress sensor and contributes to the regulation of cytoskeletal organization, cell proliferation and survival. However, whether RhoB is involved in the hypoxic response and action of glucocorticoid (GC) is largely unknown. In this study, we investigated the effects of hypoxia or/and GC on the expression and activition of RhoB in the lung of rats and human A549 lung carcinoma cells, and further studied its mechanism and significance. We found that hypoxia and dexamethasone (Dex), a synethic GC, not only significantly increased the expression and activation of RhoB independently but also coregulated the expresion of RhoB *in vitro* and *in vivo*. Up‐regulation of RhoB by hypoxia was in part through stabilizing the RhoB mRNA and protein. Inhibiting hypoxia‐activated hypoxia‐inducible transcription factor‐1α (HIF‐1α), c‐Jun N‐terminal kinase (JNK) or extracellular signal‐regulated kinase (ERK) with their specific inhibitors significantly decreased hypoxia‐induced RhoB expression, indicating that HIF‐1α, JNK and ERK are involved in the up‐regulation of RhoB in hypoxia. Furthermore, we found that knockdown of RhoB expression by RhoB siRNA not only significantly reduced hypoxia‐enhanced cell migration and cell survival in hypoxia but also increased the sensitivity of cell to paclitaxel (PTX), a chemotherapeutic agent, and reduced Dex‐enhanced resistance to PTX‐chemotherapy in A549 cells. Taken together, the novel data revealed that hypoxia and Dex increased the expression and activation of RhoB, which is important for hypoxic adaptation and hypoxia‐accelerated progression of lung cancer cells. RhoB also enhanced the resistance of cell to PTX‐chemotherapy and mediated the pro‐survival effect of Dex.

## Introduction

RhoB, a member of the family of Rho GTPases, acts as a signalling switch by cycling between an active GTP and inactive GDP (guanosine diphosphate)‐bound state. RhoB is distinguished from other Rho proteins (RhoA and RhoC) by its subcellular localization in endosomes, Golgi‐associated vesicles, and the nucleus; its rapid turnover at mRNA and protein level; as well as its post‐translational modification by the addition of lipid moieties (geranylgeranyl or farnesyl groups and palmitate) 1, 2, 3, 4, 5, 6. RhoB is involved in endocytosis and vesicle trafficking, it therefore contributes to the cytoplasmic membrane targeting of a variety of receptors and signalling molecules such as epidermal growth factor receptor, platelet‐derived growth factor receptor, non‐receptor kinases src and Akt in cells 6, 7, 8. RhoB can be rapidly up‐regulated by cytokines or growth factors, such as TGF‐β 1, 9, 10 and be activated by genotoxic stress and heat stress 11, 12, 13, 14. It is considered a stress sensor and plays an important role in the regulation of cytoskeletal organization, cell proliferation, cell survival and tumour invasiveness 13, 14, 15, 16, 17.

Hypoxia occurs during heart disease, acute and chronic vascular disease, and pulmonary disease. Hypoxia is also a typical phenomenon of solid tumours. Accumulating evidence has indicated that the hypoxic microenvironment increases cell growth, metastasis and resistance to chemo‐ and radiotherapy in tumours including lung cancer 18, 19, 20, 21. Hypoxic stress can affect several independent transcriptional regulators, of which hypoxia‐inducible transcription factor‐1 (HIF‐1) is the primary transcription factor that mediates the adaptive response to hypoxia by regulating the expression of its target genes 22. The effect of hypoxia on RhoB has been studied by different groups. However, the results are inconsistent; this suggests that the effect of hypoxia on RhoB is cell‐type dependent. For example, hypoxia did not affect the expression of RhoB in renal carcinoma Caki‐1 cells 23 and in U87 glioblastoma cells 24; but hypoxia did up‐regulate the expression and activation of RhoB in human pulmonary artery endothelial and smooth muscle cells, in which RhoB is involved in the regulation of pulmonary vascular tone and structure, and promotes development of pulmonary hypertension 15. It is unclear the effect of hypoxia on the expression RhoB in lung tissue and lung epithelial carcinoma cells. Since previous studies have also demonstrated that RhoB influences tumour cell proliferation, metastasis and resistance to chemo‐ and radiotherapy in lung adenocarcinoma 16, 17, we are interested in finding out whether RhoB mediates adaptive response to hypoxia in the lung and contributes hypoxia‐enhanced progression of lung cancer cells.

The release of glucocorticoid (GC) in response to hypoxic conditions in animals and humans has been extensively documented. Glucocorticoid plays an essential role in adaptation to hypoxic environments and homoeostatic regulation. Synthetic GCs, such as dexamethasone (Dex), have therefore been widely used to treat severe tissue damage because of hypoxia (such as hypoxia‐induced pulmonary edema and lung injury) 25, 26. Moreover, Dex is used in standard practice as a concomitant medication during chemotherapy of solid malignant tumours to reduce acute toxicity and to protect normal tissue of cancer patients against the long‐term effects of genotoxic drugs 27, 28. The effects of GC are mediated by the GC receptor (GR), a transcription factor that belongs to the nuclear receptor superfamily. On binding GC, GR can modulate gene transcription through either a direct binding with GC response element in the promoter region of target genes or interactions with other transcription factors, such as AP‐1 and NF‐κB 26, 29. Our previous study found that Dex up‐regulated the expression of RhoB in ovarian cancer HO‐8910 cells and RAW264.7 cells 30, 31. However, whether RhoB is involved in the GC roles in tissue and systemic adaptation to hypoxia and protecting cancer cells against chemotherapy is largely unknown.

In this study, we investigated the effects of hypoxia or/and Dex on the expression of RhoB in the lung of rats and human A549 lung carcinoma cells. We found that hypoxia and Dex alone or in combination up‐regulated the expression of RhoB *in vitro* and *in vivo*. Based on these results, we further explored its possible mechanisms and biological significance.

## Materials and methods

### Cell culture, cell exposure to hypoxia and treatment of reagents

Human lung adenocarcinoma epithelial A549 cells were cultured routinely in DMEM F‐12 medium containing 10% Newborn Bovine Serum (NBS; Gibco BRL, Gaithersburg, MD, USA) at 37°C in a 5% CO_2_ incubator. Before experiments, the medium was replaced with DMEM/F‐12 medium containing 10% Dextran‐coated charcoal (DCC) treated NBS to avoid possible interference with serum steroids. Then the cells were placed in an anaerobic system (Forma Scientific, Marietta, OH, USA) containing 1% O_2_, 5% CO_2_, 94% N_2_ for hypoxic exposure for different times 32 with or without Dex treatment. Cobalt chloride (CoCl_2_, St. Louis, MO, USA) – induced hypoxia model was established in A549 cells treated with 200 μM CoCl_2_ for the indicated times 33.

For other experiment, the cells were incubated with 100 nM Dex (Sigma‐Aldrich, St. Louis, MO, USA) for different periods of time. Control cells were incubated with ethanol (1‰). The p38 inhibitor SB203580 (10 μM), ERK inhibitor PD098059 (20 μM) and JNK inhibitor SP600125 (20 μM) (Sigma‐Aldrich) as well as the HIF‐1α inhibitor 400083 (Calbiochem, Darmstadt, Germany) were pre‐added 1 hr before hypoxic exposure.

### Hypoxic exposure of animals, adrenalectomy and Dex supplement

Sprague–Dawley rats, weighing from 200 to 250 g, were obtained from Shanghai SLAC laboratory animal company. The animal care facility is accredited by the Association for Assessment and Accreditation of Laboratory Animal Care. All animals were acclimatized in our animal laboratory for at least 7 days before the experiment. Hypoxic exposure, adrenalectomy and Dex supplement of rats were performed as previously described 34. Briefly, the randomly selected rats were put in a normobaric hypoxia chamber (40 l, Yangyuan Hyperbaric Oxygen Chamber Company, Shanghai, China) and flushed with 8% O_2_ (a gas mixture of 8% O_2_ and 92% N_2_) for different times (*n* = 9, per group). Adrenal glands of rats were removed by the dorsal approach. Sham animals underwent the same surgery except the adrenal glands were left intact. Adrenalectomized (ADX) rats were given 0.9% saline *ad libitum* to compensate for sodium loss after the operation and allowed to recover for 1 week. After that, the ADX rats were exposure to hypoxia or/and injected intramuscularly with 5 mg/kg bw of Dex dissolved in a 0.9% NaCl solution for 12 hrs. Control ADX rats were treated with 0.9% NaCl alone 35. Then animals were anaesthetized and killed, and lung tissue was isolated for follow‐up experiments.

### RNA extraction and real‐time quantitative RT‐PCR

Total RNA was isolated using TRIzol reagent (Invitrogen, Carlsbad, CA, USA), and 2 μg total RNA was reverse transcribed using Reverse Transcription Reagents (MBI Fermantas, Vilnius, Lithuania) following manufacturer's protocol. Quantitative real‐time PCR was performed in triplicate using SYBR Green PCR Master Mix (Toyobo, Japan) on a Mastercycler ep realplex (Eppendorf, German). The primer sequences used were as follows. RhoB (rat): 5′‐TGCTGATCGTGTTCAGTAAG‐3′ (forward) and 5′‐AGCACATGAGAATGACGTCG‐3′ (reverse). RhoB (human): 5′‐TGCTGATCGTGTTCAGTAAG‐3′ (forward) and 5′‐AGCACATGAGAATGACGTCG‐3′ (reverse). Thermal cycling conditions consisted of an initial denaturing step (95°C, 2 min.) followed by 40 cycles of denaturing (95°C, 15 sec.), annealing (56°C, 15 sec.) and extending (72°C, 45 sec.). The mRNA levels of RhoB were normalized to β‐actin (internal control) and relatively quantified using the 2^∆∆CT^ formula. Changes in gene expression were expressed as a relative fold‐increase in mRNA compared with that of control.

### Western blot analysis

The protein level in cells and tissues was determined by Western blot analysis as described previously 36. Briefly, protein extracts were separated by SDS‐PAGE, transferred to nitrocellulose membrane (Millipore, Ireland) and probed overnight with primary antibodies against RhoB (sc‐180; Santa Cruz Biotechnology, Santa Cruz, TX, USA), β‐actin (A5441; Sigma‐Aldrich Chemicals), HIF‐1α (H‐206; Santa Cruz Biotechnology), phosphorylated JNK, JNK, phosphorylated ERK, ERK, phosphorylated p38 mitogen‐activated protein kinase (MAPK) or p38 MAPK (Cell Signaling, Danvers, MA, USA). The membranes were washed three times and incubated with HRP‐conjugated secondary antibodies (1:5000; Rockland Immunochemicals, Philadelphia,PA, USA) for 2 hrs. Finally blots were detected by ECL chemiluminescence (Pierce, Rockford, IL, USA). Protein bands were quantified with ImageJ software (NIH, Bethesda, MD, USA) using β‐actin as an internal control.

### Rho‐GTP pull‐down assay

RhoB activity was measured using Rho‐GTP pull‐down assay kit according to the manusfacture's protocol (Cytoskeleton, Denver, CO, USA). Briefly, A549 cells were harvested in cell lysis buffer after various treatments. The lysates were centrifuged to pellet insoluble materials. An equivalent amounts of lysate from each sample was removed as an input control. The remaining lysate was combined with 60 μg Rhotekin‐RBD protein beads and gently rotated for 1 hr at 4°C. Precipitates were washed twice with wash buffer. Precipitates were resuspended with 30 μl SDS‐PAGE loading buffer and subjected to Western blot analysis.

### Transfection of RhoB‐siRNA

The siRNA targeting RhoB was designed and manufactured by GenePharma Co. Ltd (Shanghai, China). The sequences for RhoB‐siRNA were 5′‐UGCUGAUCGUGUUCAGUAATT‐3′. Negative control siRNA (siRNAs with sequences that do not target any gene product) was used to determine the transfection efficiency and to control for the effects of siRNA delivery. Twenty‐four hours after plating in 6‐well plates at the density of 4.0 × 10^5^ per well, A549 cells at approximately 30–50% confluence were transfected with each construct (10 nM) using INTERFERin^™^ (Polyplus transfection SA, Illkirch, France), according to the manufacture's instruction.

### Analysis of cell viability

Cells were transiently transfected with control siRNA or RhoB siRNA for 24 hrs and plated in 96‐well plates at the density of 1.0 × 10^4^ per well in triplicate for overnight. After indicated treatment, cell viability was evaluated by WST‐8 assay using Cell Counting Kit‐8 (CCK‐8; Dojindo Molecular Technologies, Inc., Kumamoto, Japan) according to manusfacture's protocol. The optical density was measured at a wavelength of 450 nm using a Labsystem multiskan microplate reader (Merck Eurolab, Dietikon, Switzerland).

### Cell migration assay

Cell migratory ability was assessed *in vitro* by transwell chambers (24‐well insert; pore size, 8 μm; Corning Inc., Corning, NY, USA). Breifly, following transient transfection for 36 hrs, A549 cells were typsined and plated onto the upper chamber at the density of 4.0 × 10^4^ per chamber in serum‐free medium. The medium supplemented with 10% serum was used as a chemoattractant in the lower chamber. Then transwell chambers were incubated in normoxic or hypoxic condition for 16 hrs. After that, the non‐migrating cells on the upper surface of the membrane were removed by a cotton swab. Cells on the lower surface of the membrane were then fixed with cold methanol and stained with 0.1% crystal violet. Cell migration was quantified by counting stained cells in five randomly selected fields at 100× magnification with a light microscope.

### Statistical analysis

Quantitative data were shown as mean ± S.D. The Student's *t*‐test was used to compare the difference between two different groups. A value of *P* < 0.05 was considered to be statistically significant.

## Results

### Hypoxia up‐regulates the expression of RhoB in rat lungs and A549 cells

**Figure 1:**
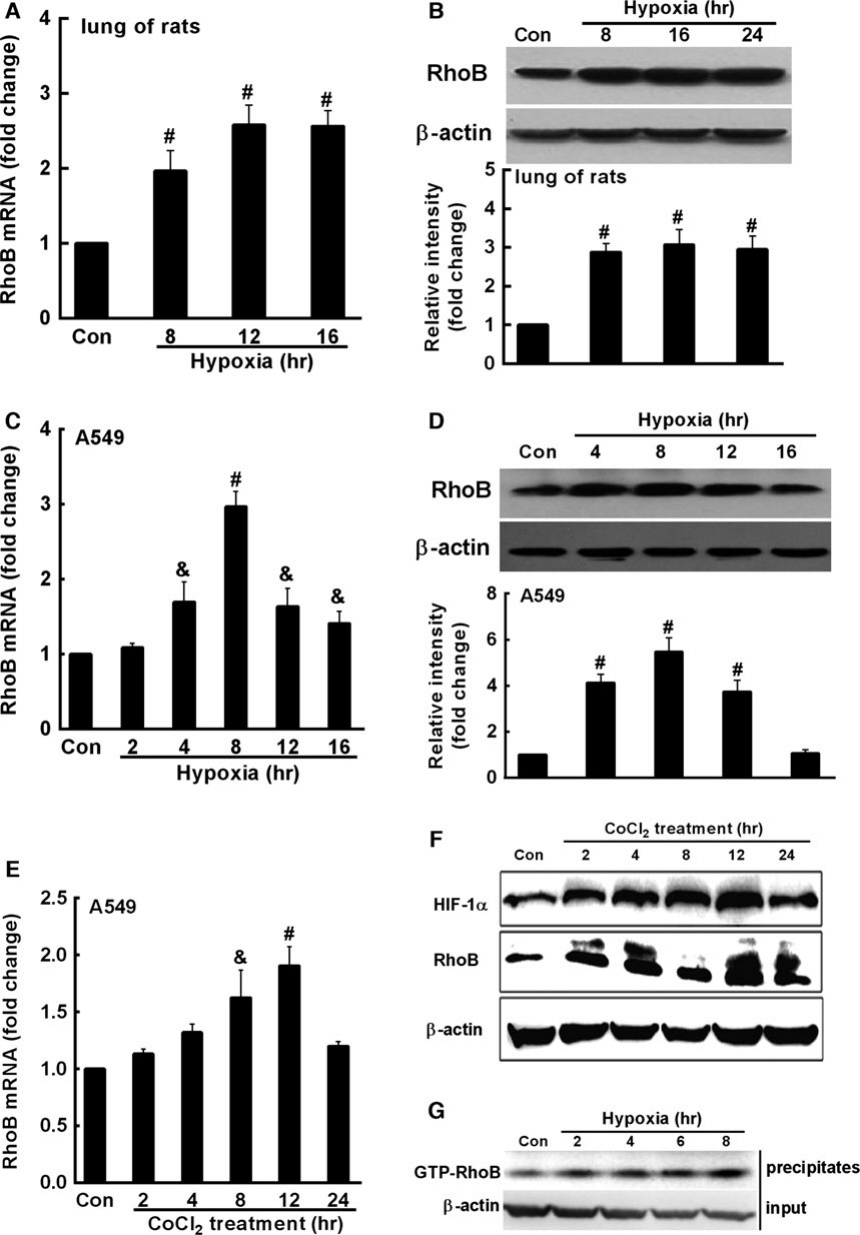
Hypoxia up‐regulates the expression of RhoB in rat lungs and A549 cells. (**A** and **B**) Adult male Sprague–Dawley rats were put in a normal pressure hypoxia chamber filled with 8% O_2_ and 92% N_2_ for the indicated times (*n* = 9, per group), and rats in the control group stayed in normoxic environment (*n* = 9). The levels of RhoB mRNA (**A**) and protien (**B**) in rat lungs after hypoxia were assessed by qRT‐PCR and Western blot. A549 cells (1.0 × 10^6^ per well) were plated for overnight and cultured in a normoxic incubator (with 5% CO_2_, 20% O_2_) or a hypoxic incubator (with 1% O_2_, 5% CO_2_, 94% N_2_) or treated with 200 μM CoCl_2_ or vehicle for the indicated times. The levels of RhoB mRNA (**C** and **E**) and protein (**D** and **F**) were assessed by qRT‐PCR and Western blot. (**G**) A549 cells (5.0 × 10^5^ per well) were cultured in 6‐well plates for overnight, then cells were exposed to hypoxia for the indicated times. RhoB activity was measured by Rho‐GTP pull down assay. The mRNA (**C** and **E**) and protein of RhoB (**D**, lower panel) were expressed as means ± S.D. of three independent experiments. Symbols & and # represent *P* < 0.05 and *P* < 0.01 compared with control cells respectively.

We first examined the expression of RhoB in the lung of rats after exposure to hypoxia (8% O_2_) for different times by quantitative RT‐PCR and Western Blot. As shown in Figure 1A and B, hypoxia significantly up‐regulated the expression of RhoB mRNA and protein with a maximal induction of RhoB protein (3.1‐fold of control, *P* < 0.01) at 16 hrs after hypoxic exposure. Hypoxic exposure (1% O_2_, Fig. 1C and D) and cobalt chloride (CoCl_2_, Fig. 1E and F), a chemical inducer of hypoxia 33, also significantly up‐regulated the expression of RhoB at mRNA and protein levels in A549 cells. The maximal increase in RhoB mRNA and protein by hypoxic exposure at 8 hrs were about 3‐ and 5.5‐fold of that of control respectively (*P* < 0.01) (Fig. 1C and D). Besides up‐regulation of RhoB expression, hypoxia (1% O_2_) also significantly increased the level of activated RhoB (GTP‐RhoB) in A549 cells (Fig. 1G).

### Hypoxia increases the stability of RhoB mRNA and protein in A549 cells

**Figure 2:**
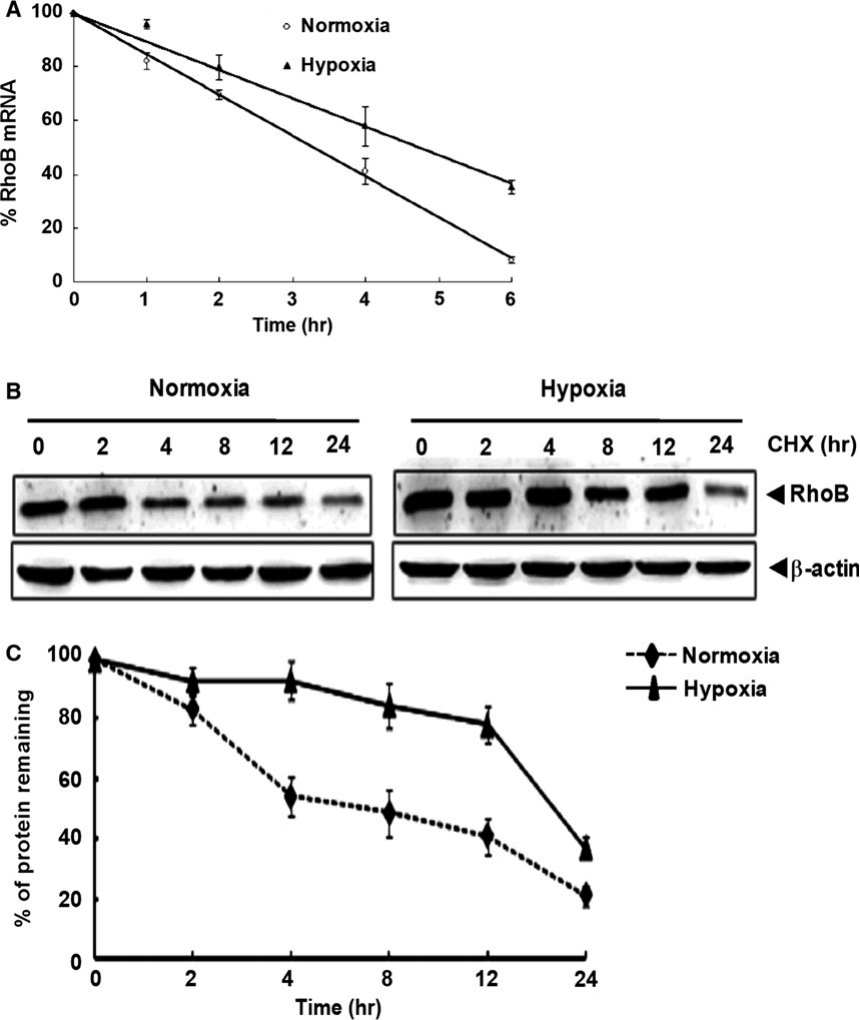
Hypoxia increases the stability of RhoB mRNA and protein in A549 cells. A549 cells (1.0 × 10^6^ per well) were plated for overnight. Following hypoxic pre‐conditioning for 8 hrs, cells were treated with actinomycin D (ActD, 5 μg/ml) or cycloheximide (CHX, 100 μg/ml) and maintained in hypoxia. Control cells were treated with ActD or CHX and maintained in normoxia. (**A**) RhoB mRNA level in cells treated with ActD was measured by qRT‐PCR. RhoB mRNA level was normalized against β‐actin and plotted as a percentage of total RhoB mRNA *versus* time. (**B**) The protein level of RhoB in cells treated with CHX was assessed by Western blot. (**C**) The band of RhoB was quantified by densitometric analysis using β‐actin as an internal control. RhoB protein level was plotted as a percentage of total RhoB protein *versus* time. The value at each time‐point represents the means ± S.D. of one independent experiment performed in triplicate.

Since above results showed that the level of RhoB protein induced by hypoxia was more rapid and higher than that of RhoB mRNA, we investigated whether hypoxia up‐regulated the expression of RhoB through increasing the stability of the RhoB mRNA and protein. A549 cells were exposed to hypoxia for 8 hrs, and then further treated with 5 μg/ml actinomycin D (ActD, a transcription inhibitor) or 100 μg/ml actinomycin D (CHX, a protein synthesis inhibitor) for different times. As shown in Figure 2A, the half‐life of RhoB mRNA significantly extended in hypoxic A549 cells from 3.28 to 4.75 hrs (a 1.45‐fold increase, *P* < 0.01). Hypoxia also significantly extended the half‐life of RhoB protein from 8 to 19 hrs (a 2.38‐fold increase, *P* < 0.01) (Fig. 2B and C). These results indicate that RhoB up‐regulation by hypoxia is at least in part because of the increased stabilization of both RhoB mRNA and protein.

### Activation of HIF‐1α, ERK and JNK are involved in hypoxia‐induced RhoB expression in A549 cells

**Figure 3:**
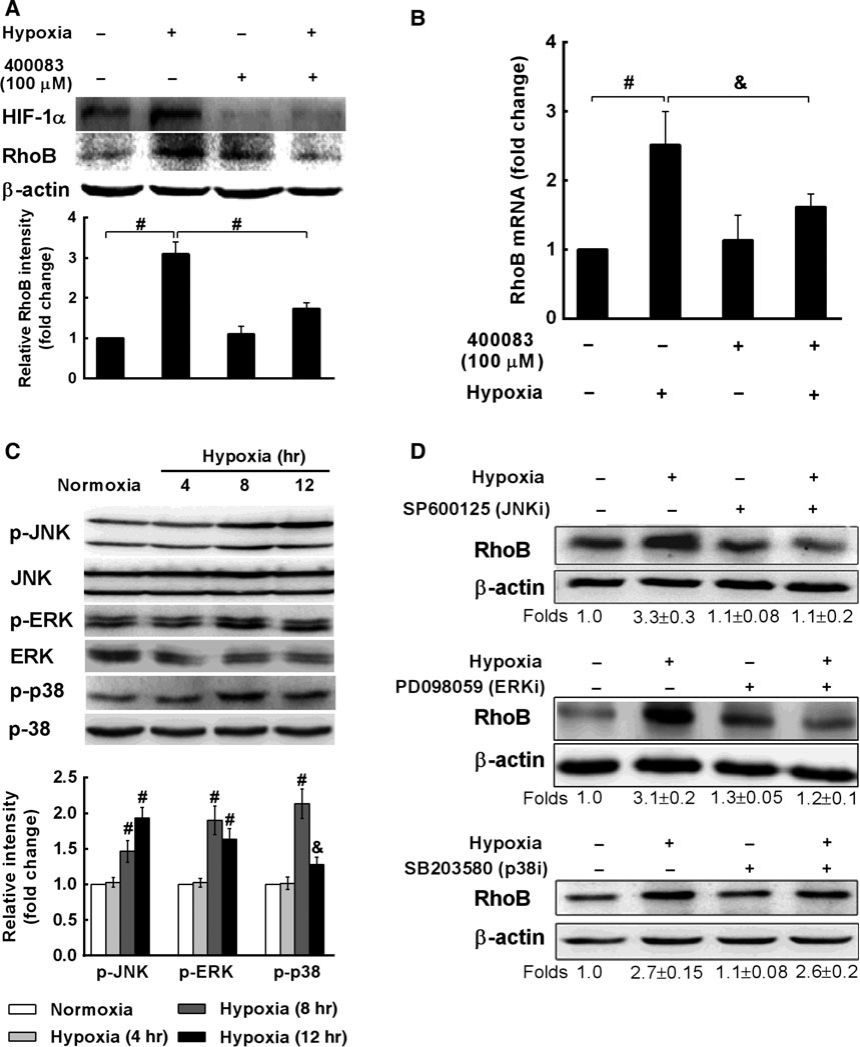
Activation of HIF‐1α, ERK and JNK, but not p38MAPK are involved in hypoxia‐induced RhoB expression in A549 cells. (**A** and **B**) A549 cells (1.0 × 10^6^ per well) were cultured for overnight and were pre‐incubated with or without HIF‐1α inhibitor 400083 (100 μM) for 1 hr prior to hypoxic exposure for 12 hrs (**A**) or 8 hrs (**B**). HIF‐1α and RhoB proteins were assessed by Western blot and β‐actin was used as a loading control. (**B**) RhoB mRNA was assessed by qRT‐PCR and β‐actin was used as a normalization control. (**C**) A549 cells were exposed to hypoxia for indicated times, then p‐JNK, JNK, p‐ERK, ERK, p‐p38 and p38 were detected by Western blot. (**D**) A549 cells were pre‐incubated with or without SB203580 (10 μM), SP600125 (20 μM) or PD098059 (20 μM) for 1 hr prior to hypoxic exposure for 12 hrs, and RhoB protein was assessed by Western blot. The protein band was quantified by densitometric analysis. The value represents mean (±S.D.) of increased folds *versus* normoxic vehicle control from three independent experiments. Symbols & and # represent *P* < 0.05 and *P* < 0.01 compared with control cells respectively.

It is known that hypoxia enhances the expression of transcription factor hypoxia‐inducible factor HIF‐1α 22 and activates several kinases and signal transduction pathways, including ERK, JNK and p38 MAPK in MAPK family 37, 38, 39, 40. However, whether HIF‐1 or these kinases are involved in hypoxia‐induced RhoB expression remains unclear. Here, we investigated the possible involvement of HIF‐1 and MAPK pathways in up‐regulation of RhoB by hypoxia. The results showed that hypoxia increased protein levels of HIF‐1α and RhoB. Treatment of A549 cells with 100 μM 400083, a specific inhibitor of HIF‐1α, not only reversed the increase of HIF‐1α but also markedly decreased hypoxia‐induced RhoB expression at protein and mRNA levels (Fig. 3A and B). We further demonstrated that hypoxia‐activated ERK, JNK and p38 MAPK by increasing their phosphorylated levels (Fig. 3C). Specific inhibitor of ERK (PD098059) or JNK (SP600125) reduced hypoxia‐induced expression of RhoB protein. However, specific inhibitor of p38 MAPK (SB203580) had no such effect (Fig. 3D). These data indicate that HIF‐1α and activated JNK and ERK (but not p38 MAPK) are involved in the induction of RhoB by hypoxia in A549 cells.

### Up‐regulation of RhoB by hypoxia promotes cell survival and migration in A549 cells in hypoxia

**Figure 4:**
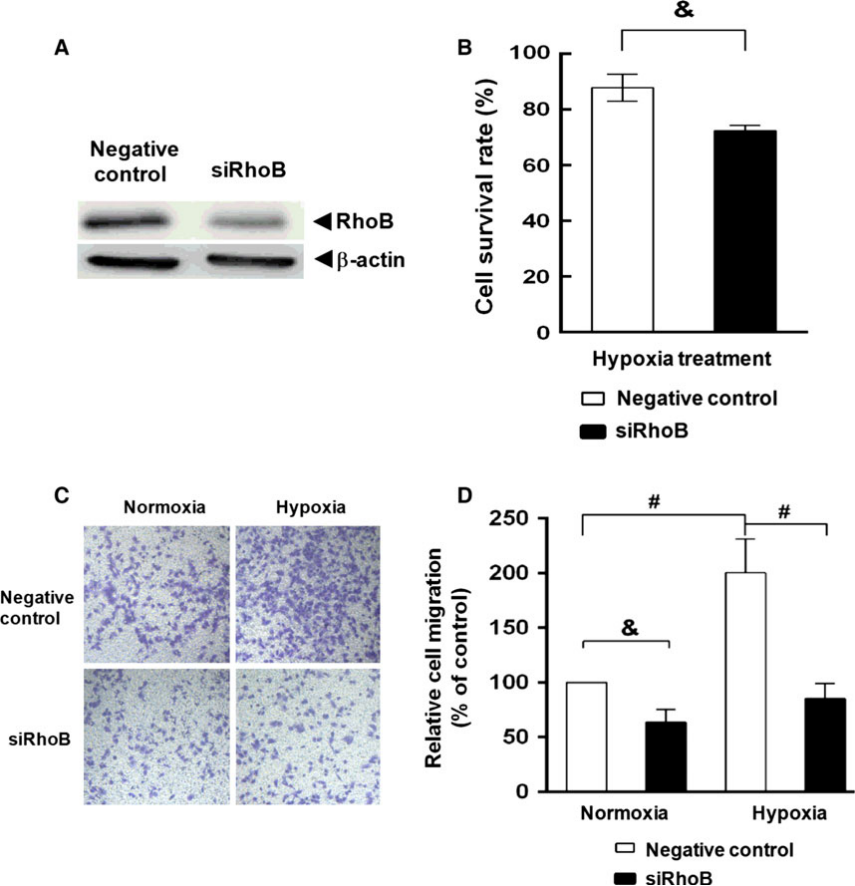
Up‐regulation of RhoB by hypoxia promotes cell survival and migration in A549 cells in hypoxia. A549 cells were transiently transfected with RhoB siRNA or negative control siRNA for 24 hrs. (**A**) RhoB protein was detected by Western blot. (**B**) A549 transfectants were exposed to hypoxia for 24 hrs, then cell viability was assayed by WST‐8 assay as described in ‘Materials and methods’. (**C** and **D**) Migratory ability of A549 transfectants was measured by transwell (Boyden Chamber) assay following transient transfection for 36 hrs as described in ‘Materials and methods’. (**C**) Image was a representative of three independent migration assays. Values were expressed as mean (±S.D.) from three independent experiments. Symbol & and # represent *P* < 0.05 and *P* < 0.01 respectively.

Previous studies have indicated that there is a high correlation between hypoxia and lung cancer metastasis and poor prognosis 20, 21. Therefore, we investigated the significance of up‐regulation of RhoB in A549 cells using specific RhoB‐RNA interference (siRhoB). The results showed that hypoxic exposure for 24 hrs decreased cell viability in control A549 transfectant. Inhibiting RhoB expression with siRhoB further decreased cell viability (from 87% to 72%, *P* < 0.05) in hypoxia (Fig. 4A and B). Moreover, hypoxia markedly promoted the migration of A549 cells as previously reported 41. Inhibiting RhoB expression not only resulted in about 40% of decrease in migratory ability in normoxia but also abolished hypoxia‐enhanced cell migration (Fig. 4C and D). These results indicate that up‐regulation of RhoB by hypoxia not only increases cell viability under hypoxic condition but also plays an important role in hypoxia‐enhanced cell migration in A549 cells.

### Dex also induces RhoB expression and increases RhoB activity in A549 cells

**Figure 5:**
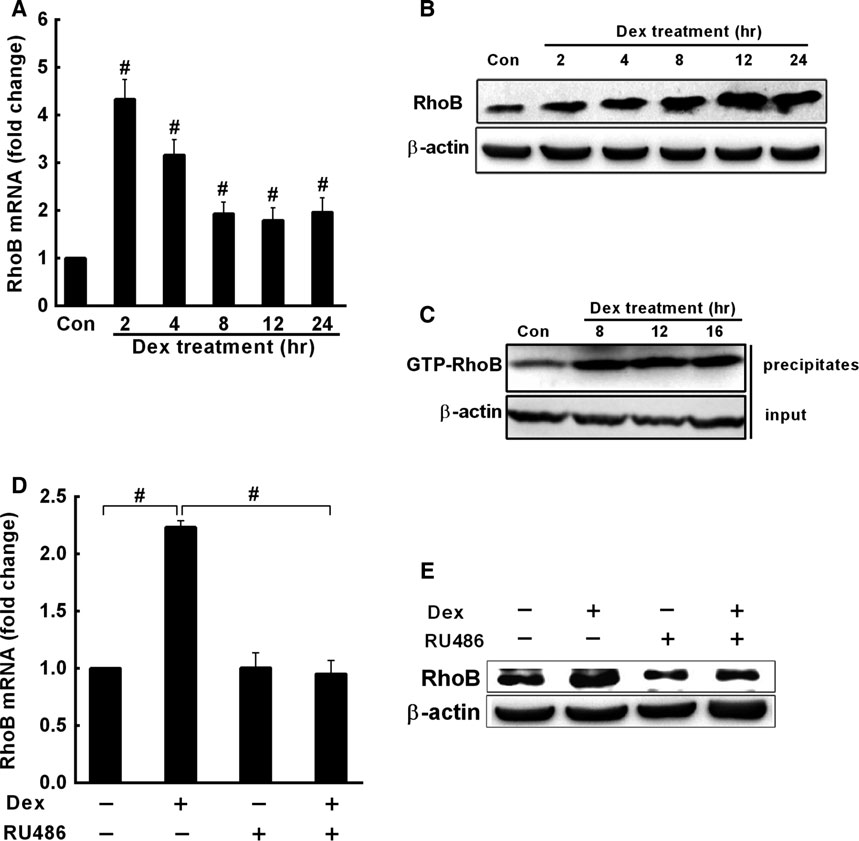
Dex induces the expression of RhoB and increases RhoB activity in A549 cells. (**A** and **B**) A549 cells (5.0 × 10^5^ per well) were maintained in 10% DCC medium for 12 hrs and treated with either ETOH vehicle or 100 nM Dex for the indicated times. The levels of mRNA (**A**) and protein (**B**) of RhoB were assessed by qRT‐PCR and Western blot analysis respectively. (**C**) RhoB activity was measured by Rho‐GTP pull down assay. Cells (5.0 × 10^5^ per well) were maintained in 10% DCC medium for 12 hrs and treated with either ETOH vehicle or 100 nM Dex in the absence or presence of 100 nM RU486 for 12 hrs. RU486 was added 1 hr prior to the addition of Dex for antagonism analysis. The expression of RhoB mRNA and protein were detected by qRT‐PCR (**D**) and Western blot analysis (**E**) respectively. The value represents mean (±S.D.) of increased folds *versus* vehicle control from three independent experiments. # represents p < 0.01.

Since hypoxia results in releasing high levels of endogenous GC, which plays an important role in hypoxic adaption *in vivo*, we examined the effect of Dex, a synethic GC, on the expression of RhoB in A549 cells. The results demonstrated that 100 nM Dex rapidly increased the mRNA and protein level of RhoB with the maximal induction of RhoB mRNA (4.3‐fold of control, *P* < 0.01) at 2 hrs after Dex treatment (Fig. 5A and B). Dex also significantly increased RhoB activity in A549 cells (Fig. 5C). Moreover, the RhoB mRNA and protein induced by Dex were almost blocked by RU486, an antagonist of GR (Fig. 5D and E), indicating that up‐regulation of RhoB by Dex is mediated by GR.

### Up‐regulation of RhoB enhances cell resistance to chemotherapeutics and is involved in the pro‐survival effect of Dex in A549 cells

**Figure 6:**
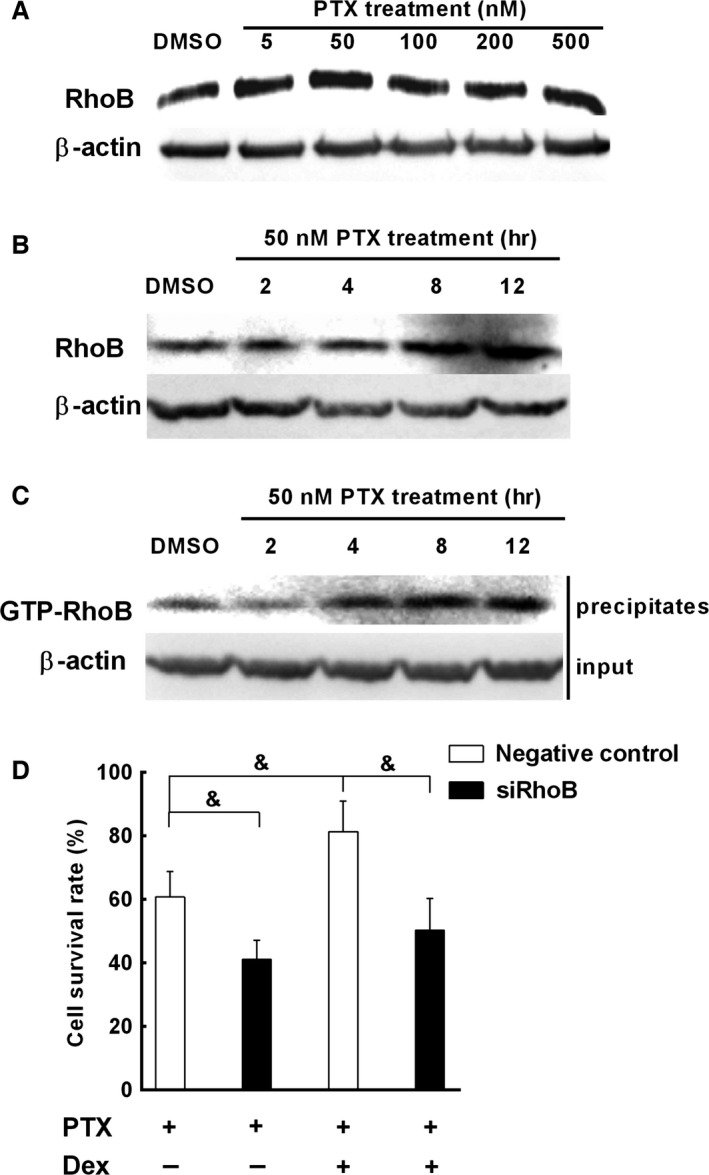
Up‐regulation of RhoB is involved in chemotherapeutic resistance and pro‐survival effect of Dex in A549 cells. A549 cells (5.0 × 10^5^ per well) were cultured for overnight, then cells were treated with different concentration of PTX for 8 hrs or 50 nM PTX for the indicated times. (**A** and **B**) RhoB protein was detected by Western blot. (**C**) RhoB activity was measured by Rho‐GTP pull down assay. β‐actin was used as an internal control. (**D**) A549 transfectants were treated with 500 nM PTX alone or cotreated with 100 nM Dex for 24 hrs, then cell viability was assayed by WST‐8 assay as described in ‘Materials and methods’. Values were expressed as mean (±S.D.) from three independent experiments. Symbol & represents *P* < 0.05 *versus* control transfectants.

Paclitaxel (PTX) is widely used as an antimicrotubule agent for the treatment of lung cancer. Unfortunately, the resistance to this antimicrotubule agent occurs frequently. Dex is typically administered the day before, the day of and the day after chemotherapy of non‐small cell lung cancer (NSCLC), and could protect tumour cells against the cytotoxicity of chemotherapeutic agent 28. Therefore, we further investigated whether the up‐regulation of RhoB expression increased the resistance of A549 cells to PTX and contributed to the cytoprotective effect of Dex in A549 cells. We found that besides hypoxia and Dex, PTX also significantly induced the expression of RhoB (Fig. 6A and B) and increased RhoB activity in A549 cells (Fig. 6C). Inhibiting RhoB expression by siRhoB not only increased cell sensitivity to PTX but also significantly reduced the pro‐survival effect of Dex in the presence of PTX (Fig. 6D). These results indicate that up‐regulation of RhoB enhances the resistance of A549 cells to PTX and is involved in the effect of Dex‐enhanced cell resistance to chemotherapeutics.

### Dex and hypoxia have additive effects on up‐regulation of RhoB expression *in vivo* and *in vitro*

**Figure 7:**
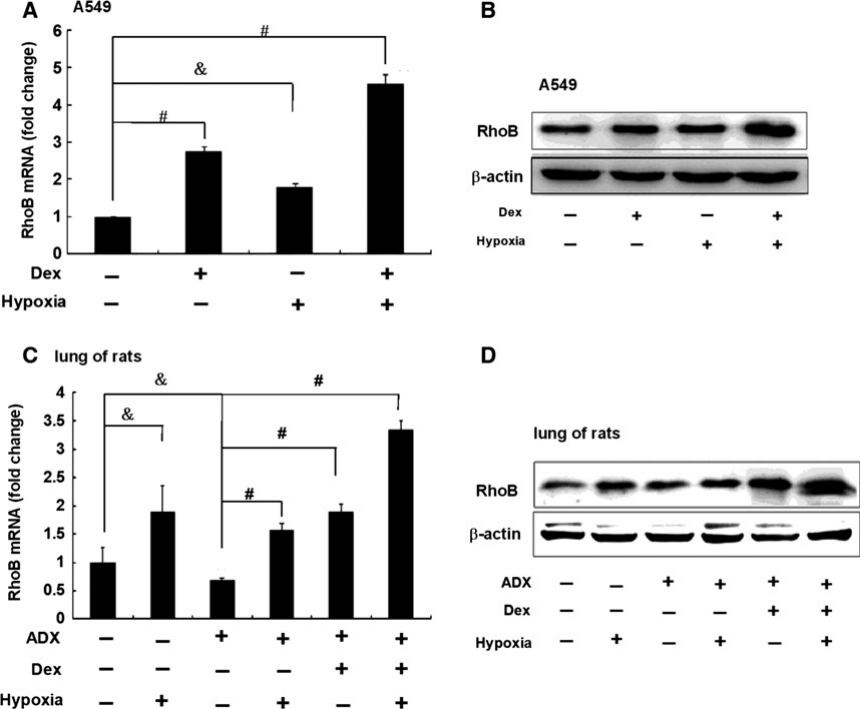
Dex and hypoxia have additive effects on up‐regulating RhoB expression *in vivo* and *in vitro*. (**A**) A549 cells (2.0 × 10^5^ per well) were cultured in 12‐well plates in triplicate for overnight and treated with 100 nM Dex or hypoxia alone or both together for 4 hrs for detection of RhoB mRNA level. (**B**) A549 cells (5.0 × 10^5^ per well) were cultured in 6‐well plates for overnight and treated with 100 nM Dex or hypoxia alone or both together for 8 hrs for detection of RhoB protein level. (**C** and **D**) Adult male Sprague–Dawley rats underwent adrenalectomy (ADX), then stayed in normoxia (*n* = 6) or hypoxia chamber filled with 8% O_2_ and 92% N_2_ for 12 hrs (*n* = 6), intramuscular injected with Dex (5 mg/kg) alone (*n* = 6) or together (*n* = 6). Sham rats were treated with or without hypoxia (*n* = 6, per group). The levels of RhoB mRNA (**C**) and protein (**D**) in the lung tissue were assessed by qRT‐PCR and Western blot. Results were representative of three independent experiments. The results of RhoB mRNA were expressed as mean ± S.D. Symbols & and # represent *P* < 0.05 and *P* < 0.01 respectively.

We examined the effect of Dex alone or in combination with hypoxia on the expression of RhoB in A549 cells. As shown in Figure 7A, Dex and hypoxia alone increased RhoB mRNA to 2.77‐fold and 1.81‐fold compared to that in control cells respectively. Cotreatment of cells with hypoxia and Dex further increased the mRNA level of RhoB (4.55‐fold of that in control cells, *P* < 0.01). Similar results were observed at RhoB protein levels of A549 cells (Fig. 7B).

Next, we investigated the effect of hypoxia or/and GC on the expression of RhoB in the lung of ADX rats in which endogenous adrenal hormones (mainly GC) were removed. As shown in Figure 7C, RhoB mRNA in ADX group was decreased to about 68% of that in sham group in normoxia. Hypoxia significantly up‐regulated the expression of RhoB mRNA in sham group and ADX group (2.4‐fold of that in the ADX control group, *P* < 0.01). Moreover, the administration with Dex (5 mg/kg) in ADX rats for 12 hrs significantly increased the expression of RhoB mRNA to 2.8‐fold of that in the ADX group in normoxia (*P* < 0.01), and further increased RhoB mRNA expression to fivefold of that in the ADX group in hypoxia (*P* < 0.01). Similar results were observed at RhoB protein levels in different groups (Fig. 7D). These results indicate that not only hypoxia or/and Dex alone up‐regulate the expression of RhoB, but also they co‐induce the expression of RhoB *in vitro* and *in vivo*.

## Discussion

Other studies and our previous work have revealed that RhoB is a DNA damage‐ and heat stress‐inducible gene 11, 12, 13, 14. In this study, we found that hypoxia not only significantly up‐regulated the expression of RhoB at mRNA and protein levels in the lung of rats and A549 cells but also increased the level of activated RhoB (GTP‐RhoB). RhoB up‐regulation by hypoxia is at least partly because of the increased stability of both RhoB mRNA and protein through post‐transcriptional and post‐translational mechanisms. Further experiments showed that hypoxia‐induced HIF‐1α contributes to the up‐regulation of RhoB in A549 cells. Although HIF‐1α plays a central role by regulating the expression of its target genes in hypoxia 22, we did not find the classical HIF‐responsive element in the promoter region (3 kb) of the RhoB gene using several transcription factor‐binding site prediction softwares; this supports the idea that HIF‐1α may up‐regulate the expression of RhoB at a post‐transcriptional level. In addition, hypoxia‐activated ERK and JNK, but not p38MAPK are also involved in the up‐regulation of RhoB by hypoxia. Exactly how HIF‐1α, ERK and JNK up‐regulates the expression of RhoB is unclear. The underlying mechanism needs to be further elucidated.

Several studies have reported that RhoB plays an important role in protecting cells from stress‐induced cell death 13, 14. Here, we further demonstrated that RhoB promotes cell survival in hypoxia. In addition, we found that besides DNA‐damaging drugs, PTX, an antimicrotubule drug used to treat cancers, also increased the expression and activation of RhoB. Knockdown of RhoB significantly decreased cell viability during treatment of PTX, indicating that RhoB enhances cell resistance to PTX‐chemotherapy in lung adenocarcinoma cells. The cytoprotective effects of RhoB may occur through multiple mechanisms. Our previous study found that overexpression of RhoB significantly enhanced transcriptional activity of NF‐κB in unstressed HO‐8910 cells and heat stressed A549 cells 14, 30. NF‐κB plays a key role in the modulation of cell survival and apoptosis by regulating the expression of several anti‐apoptotic and pro‐survival genes such as cIAP2, Bcl‐2, Bcl‐xL and Survivin 42, 43. Other groups have reported that RhoB protects human keratinocytes from UVB‐induced apoptosis through the activation of the PI‐3K‐AKT pathway, which is another important pro‐survival pathway 13. Moreover, here we found that HIF‐1α contributes to the up‐regulation of RhoB by hypoxia in A549 cells, whereas Skuli, *et al*., reported that RhoB stabilizes the HIF‐1α in U87 glioblastoma cells 24. These results suggested that there might be a positive feedback loop between HIF‐1α and RhoB in hypoxia response, which is important for mediating the adaptation and tolerance of cells under a hypoxic microenvironment.

Emerging lines of evidence suggest that hypoxia in solid tumours including lung adenocarcinoma facilitates tumour metastasis and enhances their resistance to chemo‐ and radiotherapy 18, 19, 20, 21. However, it is unclear whether RhoB is involved the effect of hypoxia on lung cancer. We demonstrated that hypoxia‐induced RhoB promoted cell survival and played an important role in hypoxia‐enhanced cell migration. These results are consistent with a recent report that ablation of RhoB levels in lung adenocarcinoma tumour cells dramatically decreased bone metastasis in an experimental model, while RhoB overexpression was associated with enhanced tumour dissemination from the primary location and increased resistance to chemotherapy and radiation 17. Therefore, it appears that hypoxia‐induced RhoB is involved in hypoxia‐enhanced progression and metastasis of lung cancer cells.

Glucocorticoids were reported to promote the survival of tumour cells and protect them against chemotherapy‐induced cell apoptosis including NSCLC cells, which has important clinical relevance when they interfere with the effect of chemotherapeutics 27, 28. The role of GC is mediated by GR through regulating several pro‐survival and anti‐apoptotic genes, such as cIAP‐2, Bcl‐XL, Bcl‐2, mitogen‐activated protein kinase phosphatase‐1, as well as GC‐induced serum, and GC‐inducible kinase‐1 44, 45, 46. In this study, we found Dex not only up‐regulated the expression of RhoB independently but also co‐induced the expression of RhoB with hypoxia *in vitro* and *in vivo*. Up‐regulation of RhoB promoted cell survival and contributed to Dex‐enhanced cell resistance to PTX‐chemotherapy in A549 cells.

**Figure 8:**
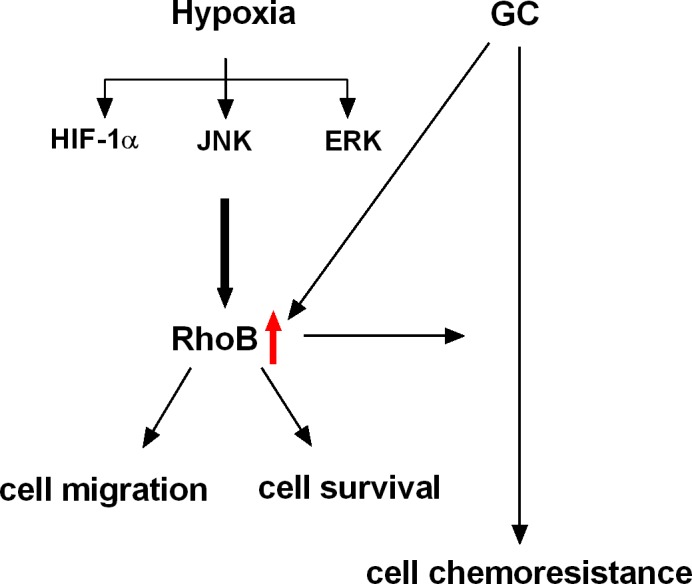
A proposed model for the mechanism and significance of up‐regulation of RhoB expression by hypoxia and glucocorticoid. This schema summarized the main signallings by which hypoxia and glucocorticoid up‐regulated the expression of RhoB and the roles of RhoB in hypoxic adaptation and hypoxia‐induced progression of lung cancer.

Taken together, this study found that hypoxia and Dex not only increased RhoB expression and activity independently but also coregulated the expresion of RhoB in rat lungs and A549 cells. Hypoxia‐induced HIF‐1α as well as activated ERK and JNK pathways were involved in up‐regulation of RhoB expression. Up‐regulation of RhoB enhanced cell migration in hypoxia, promoted cell survival and was involved in Dex‐enhanced cell chemoresistance (Fig. 8). The data provide a novel evidence that RhoB is important for cellular adaptation to hypoxia and hypoxia‐induced progression of lung cancer.

## Disclosure

The authors have no financial conflicts of interest.
